# Fractures

**Published:** 1879-08

**Authors:** 


### FRACTURES.

When a bone is broken get it as near as may be in its natural position, and then send for the doctor. Meantime wet cloths or cotton in water as warm as can be borne comfortably and apply over or around the fractured portion to prevent swelling.

Simple cases of fracture are not urgent. Some surgeons prefer to wait several days until all swelling is reduced before setting the bones.

